# Relational Reasoning and Educational Applications

**DOI:** 10.3390/bs16020210

**Published:** 2026-01-31

**Authors:** Lindsey Engle Richland, Hongyang Zhao

**Affiliations:** 1 School of Education, University of California, Irvine, Irvine, CA 92697, USA; 2 Department of Educational Psychology, College of Education, University of Illinois Urbana-Champaign, Champaign, IL 61820, USA; zhaohy@illinois.edu

**Keywords:** relational reasoning, analogy, learning

## Abstract

As the global economy and sociopolitical, technological, climate, and international trade contexts continue to shift rapidly, educational goals and aims for skilled employment are changing accordingly. Research on the cognition of relational reasoning provides insight into training youth to handle these types of complex systems and multi-faceted problems. Relational reasoning is the ability to make inferences from the relationships within and between mental representations, and grounds many higher order cognitive actions such as generating solutions to novel problems, transferring insights from one context to another, and making creative leaps of inference that are central to these societal aims. This article synthesizes the theoretical and foundational literature to highlight specific practices that are relevant and implementable in enhancing all students’ access to rich and complex thinking and reasoning skills. These include strategies for training students to notice and attend to relational correspondences, to use visual stimuli and language to draw connections, and to reduce the cognitive resources required to do so.

“*As a nation, we’ve reached at least a rhetorical consensus that our societal health requires us to provide all of our students with the sort of “thinking curriculum” we long reserved for a small elite—and accomplishing this will require a greater commitment to equitable education than ever before*”

(Darling-Hammond, 2022)

## 1. Introduction

With the rapid pace of technology growth, artificial intelligence (AI), and the nature of the interconnected global economy, economic and educational aims are shifting, leading some to describe the need for Education 4.0—a new vision of education that moves away from educational norms rooted in industrial, factory-style employment to a greater focus on the ability to think critically, innovate, evaluate, and integrate ideas and fields of knowledge for new purposes (World Economic Forum, 2023). Thus, increasingly, educational goals center on cognitive skills, including the ability to think creatively, generate solutions to novel problems, weigh evidence to make judgements, and debate solutions to problems to determine most efficient and effective approaches (see OECD, 2025).

In parallel, then, employers and researchers alike have been positing that the format of schools must reflect these new goals, including updating the “factory model” of education in which discrete, topically focused tasks are given by each teacher in a series of stages throughout the day (Darling-Hammond et al., 2024). K-12 educational aims have been moving away from this emphasis to a greater focus on the abilities to think critically, evaluate, and integrate ideas and fields of knowledge for new purposes, but what this entails for practice is not easily translated. School education standards and traditions have largely centered on clarifying the discrete series of topics that must be covered in an academic year or providing support for topically focused lesson plans.

This shift has provided new opportunities for cognitive psychological research to engage with educational practice, providing insight into how humans think, reason, and how these develop from childhood to adulthood. This article reviews research specifically on the development of relational and analogical reasoning: broadly, the ability to reason about relationships rather than the perpetual appearance of objects (Gentner, 1983). More precisely, we focus on analogy, which is a process of structured relational thinking that requires representing phenomena as systems of relationships that are then used to make inferences about the world from the relationships within and between these mental representations (Gentner et al., 2001). While seemingly abstract, the affordances of this type of reasoning apply across content areas of the traditional K-12 educational curriculum. In this review, we aim to provide novel insights into the way in which the literature on relational reasoning may inform best practices for pedagogy.

Incorporating more intentional uses of relational reasoning during instruction may be key to improving the equity and overall success of 21st century education—enabling all students to access and learn from a “thinking curriculum” (Darling-Hammond, 2022). Relational reasoning is core to innovation and creativity (Markman & Wood, 2009), as well as learning with understanding and the potential for transfer (see Gentner, 2003; Richland & Simms, 2015; Skemp, 1979).

In order to help highlight the utility and opportunities available by drawing on the relational reasoning literature to improve pedagogical practice, this article^1^ will review the foundational and contemporary theories for how humans learn to reason relationally, and highlight ways that the learning context can be shaped to improve the likelihood that children can and do reason successfully. This should facilitate both learning for content and strengthening reasoning skills. We also highlight an important distinction that has emerged between *ability* to reason relationally and *likelihood* of doing so; this distinction means that, in order to lead children to leave school as thinkers who will actively engage with and reason adaptively with their environment, schools not only need to teach children to improve their reasoning skills but also must socialize them to seek and use opportunities to do so (Richland & Simms, 2015).

## 2. Defining Relational Reasoning for Educational Contexts

When a child is able to point to a location on a tree if asked “If a tree had a knee, where would it be?” (Gentner, 1977a, 1977b), they are doing something incredibly profound and challenging. Though this comes so naturally to humans that it sometimes goes under-appreciated, such an action requires a mental representation of a human to be made, for the person to draw on stored knowledge of the term “knee”, and for them to conceptualize the relative location of a knee to the rest of the body. Then, they must align that representation to something that looks different, is spatially distinct, that does not functionally use a “knee” and indeed has no mobility, and likely is not something they have ever thought of together with a human body before. After doing both, the child must take their mental representation of this spatial relationship within the human to make inferences about the relatively same spatial location between a “knee” and the full height of the tree. Thus, this process of drawing on relational similarity between these representations includes multiple steps.

Reasoning with higher-order relations involves first including the construction of relational representations (of a human/knee and a tree), finding alignment and relational similarity between representations, mapping between the relational correspondences, and making inferences based on these correspondences (Holyoak & Thagard, 1989; Gentner, 1983). This can be formalized as identifying a relationship or system of relationships with representation 1, “A:B”, and aligning these to the similar relationship or system of relationships within representation 2, “C:D.”

A useful connection between the basic research literature on relational reasoning and more contemporary studies of classroom learning has been their highlighting the role of higher-order thinking in relational reasoning. Higher-order thinking is a potent mode of learning (see Frausel et al., 2020; Lewis & Smith, 1993), characterized by structured relational reasoning in which a learner draws a higher-order relationship between two lower order relationships or systems of relationships (Gentner, 1983). This might be “same”, which means we can take what we know about one situation and apply it to another context—which would be a classic analogy. This type of higher-order thinking in a school setting would allow for instruction about one type of math problem to be transferred to solve a second math problem. Or, it could allow a student to examine two primary documents in history to recognize that the same pattern of a phenomenon is happening in both contexts (see Richland & Simms, 2015, for further discussion).

Reasoning that centers on inferences derived from finding the same higher-order relation between these relational correspondences is generally defined as analogical reasoning, with the formalization depicted as the relations between A and B are equivalent to those between c and d. A:B = C:D (Gentner, 1983). Though, empirical research centered in everyday contexts has demonstrated that, when executed in a real-world context, unless the two representations are identical, there are also differences inherent in these relational alignments—thus, in order to effectively align a spatial relationship such as “knee” across a human and tree, one must also consider and grapple with the differences between a tree and a human, such as the overall size and movement patterns (see Richland et al., 2004). This can be true when scientists try to interpret unexpected research findings (Dunbar, 1997) or when mathematics students aim to distinguish between problems that appear similar but have important differences (e.g., between a solvable inequality and one with no solution).

Cognitively, in order to conduct higher-order relational reasoning, one must also find and create mental representations of the lower-order relations (e.g., A:B; see Doumas & Hummel, 2013). These lower-order relations may involve simple relations, such as “bigger than”. These simple relations often mask larger relational systems, however, as one can see if we consider a causality relation. One may infer and state that “A causes B”, and then, upon further reflection, it may become clear that this is actually a higher-order relation between two relational systems: “A” here might comprise a system of relations that together cause “B.” For example, the statement that “climate change may cause reduced rainfall” involves a complex system of relationships causing another complex system of relationships.

So, for the current purposes, we will focus on reviewing the literature on the ways in which children represent and reason about higher-order relations, and the implications of that work for supporting education; however, we see this as an overarching reasoning category that includes other levels and aspects of reasoning. Other forms of relational reasoning include making inferences other than structural similarity equivalence from relational correspondences, such as antithesis (Alexander & The Disciplined Reading and Learning Research Laboratory, 2012).

### 2.1. Structure Mapping in Higher-Order Thinking

The structure mapping model of analogy provides a framework for considering the cognition involved in reasoning relationally, which is highly informative for conceptualizing how to best support children in acquiring these skills, as well as to develop and test actionable pedagogical practices that align with extant learning data. Structure mapping refers to the process of creating a mental representation of relationships within a source and target object and aligning these representations in order to make inferences (Gentner, 1983). For example, if one is learning about patterns in climate change, one might make a mental representation of the relations within the range of temperatures in Phoenix, Arizona, and those in Chicago, Illinois, to find that both are experiencing a broader range than in prior decades, though the ranges themselves are different. Making this alignment allows one to make a broader inference that climate change is yielding broadening temperature swings across ecosystems.

Importantly, however, it is also the case that the pragmatics of one’s larger goal-oriented reason for making structural alignments may lead to different relational representations of a source and target, and also different alignments and mappings between the representations, as highlighted by the Multi-Constraint model of Analogy (Holyoak & Thagard, 1989; Spellman & Holyoak, 1992). For example, a reasoner might represent the same range of temperature values in the same two cities described above as both demonstrating higher and lower temperatures than in previous decades, thus leading to the inference that climate change is not yielding broader global warming.

Thus, it is crucial to understand that, in order to use relational reasoning effectively in educational contexts, instructors must recognize that learners will not always create the same mental representations intended by the instructor who by definition is more of an expert in the field, and the pragmatics and pedagogical context of the opportunities for reasoning must be crafted intentionally (Simms & Richland, 2019). If not, there are ample opportunities for producing misconceptions or misunderstandings by learners (Zook & Di Vesta, 1991).

### 2.2. Aligning a Structure Mapping Model of Relational Reasoning with Educationally Defined Higher-Order Thinking

While the structure mapping model has generated insights for educational practice which we will review below, they are often very theoretically precise but use different vocabulary and meaning than those used by educators. Thus, we draw connections between this literature and principles of “higher-order thinking,” defined as forms of thinking that necessarily involve inferences that go beyond the given information, building adequate representations, and analyzing and constructing relations (Lewis & Smith, 1993; Resnick, 1987). A common feature of higher-order cognitive functions is that they are invoked in novel situations where the simple recall or direct application of existing mental structures—typically associated with lower-order thinking—no longer suffices to achieve the desired goal (Lewis & Smith, 1993). The authors elaborated, “higher-order thinking occurs when a person takes new information and information stored in memory and interrelates and/or rearranges and extends this information to achieve a purpose or find possible answers in perplexing situations (p. 28).” To bridge the gap between the “unknown” (i.e., the desired goal or the solution to the novel problem) and the “known” (i.e., existing knowledge structure), individuals often rely on structural mapping, the core mechanism underlying relational reasoning. Through this process, learners seek both similarities and differences at the structural level in order to adapt prior solutions to novel problems.

Consistent with this view, Bloom’s taxonomy of educational objectives (Anderson & Krathwohl, 2001; Bloom, 1956) emphasizes the importance of measuring learning through cognitively demanding tasks (higher order thinking tasks) such as application, analysis, synthesis, and evaluation. Importantly, all of these involve cognitive processes of relational thinking Viewing learning content as coherent yet decomposable systems of relations, which is the first step or prerequisite of structural mapping, allows for insight into the cognition underpinning many of these types of tasks. For example, to apply knowledge to novel math problems and to analyze, synthesize, and evaluate math problem solutions, students must conceptualize mathematics as a system of quantitative relations governed by reasoning, rather than isolated principles to be memorized. Moreover, mapping these relations across structurally similar systems can promote creative problem solving and flexible transfer, both of which are hallmarks of deep conceptual understanding. In this way, structural mapping functions not only as a mechanism of relational reasoning but also as a foundational driver of broader higher-order thinking skills.

Importantly, these have different functions as well, with some uses of relational reasoning improving students’ learning for categories or concepts, while others help to produce problem-solutions and to generate creative innovations to meet real-world problems. We will next clarify different functions for relational reasoning that are relevant to educational contexts, followed by a review of literature demonstrating that there may be differences in individuals’ mindsets that lead them to notice or attend specifically to relations, which may in turn have implications for future learning quality (Zhao et al., 2025a). Finally we provide a description of specific practices that leverage the literature and that are relevant and implementable to encourage relational mindsets and structure-mapping processes in everyday learning settings. These include pedagogical strategies for training students to notice and attend to relational correspondences, including the use of relational language, and practices that instruct by using visual stimuli and language, as well as to reduce the cognitive resources required for students to notice and use relational correspondences.

### 2.3. Key Functions for Relational Reasoning in Education

Structure mapping is an underpinning mechanism for aligning and drawing inferences based on comparing representations, but the ultimate learning benefits of these relational reasoning instances can be quite varied. We describe two key functions of relational reasoning in educationally relevant learning: acquiring relational categories and using relational reasoning for problem solving and transfer.

#### 2.3.1. Relational Category Acquisition

Relational reasoning underlies the human ability to understand and infer relational concepts or categories (Kalra & Richland, 2022), which are concepts defined not by any specific property of individual items but by the network of relationships that they are part of. For example, words such as “neighbor” or “barrier” derive their meaning from the roles they play within a broader structure or the interactions they have with other components. Moreover, words like “mutually exclusive,” “opposite,” and “homogenous” directly describe the abstract relation between two or more objects. This contrasts with feature-based categories, like “apple,” “cat,” or “chair,” which are primarily defined by their intrinsic, observable attributes, such as shape, color, or function.

One challenge (and opportunity) in learning relational categories is that these words can be used to describe various entities that do not share salient similarity in the surface-level features. A “neighbor” can be a young lady who lives next to your house, or two nations that are adjacent to each other. Similarly, a “barrier” can be a fence that stops you from entering a house, or a challenging situation that prevents you from being promoted. To learn these relational concepts or categories, or to engage in relational reasoning, children need to move beyond the obvious, observable dissimilar surface-level features, and instead engage in structural comparisons where they need to juxtapose and hold at least two sets of entities in their mind (e.g., two houses next to each other and two nations adjacent to each other), align them based on the role that each entity plays within each set, and abstract or infer the commonality in the relation that the two entities form within each set. Finding the higher-order “same” relationship between two instances of a lower-order relation, i.e., “adjacent” or “neighbor,” is a potent way to generalize that the relation can apply to other contexts that share the same relational properties, (i.e., two families living next to each other or two nations adjacent to each other). The work of finding higher-order relations, or “relations among relations,” can support abstraction and category learning (Gick & Holyoak, 1983; also see Alexander & The Disciplined Reading and Learning Research Laboratory, 2012; Dumas et al., 2013).

The process of noticing and learning relational concepts is more cognitively demanding than noticing a single shared property (e.g., all cats are small, furry, four-legged animals). In contrast, relational concept acquisition requires abstraction, which is effortful but at the same time provides the opportunity to apply these concepts to a broader range of contexts. Such alignment is hypothesized to derive from aligning multiple representations of relations, inhibiting attention to features that are not alignable (e.g., humans versus geographical regions) to abstract out the relations that are common (e.g., “adjacent”) (see Gick & Holyoak, 1983). Once abstracted, this schema becomes easier to apply to new contexts or problem-solving situations. Also importantly there may be single levels of complexity to these correspondences and relations (two adjacent nations) or there may be increasing levels of complexity, which increase the working memory demand as well as the level of complexity of the distraction (e.g., a higher-order relation “same” may involve mapping a single level of complexity—as in “two objects are adjacent” or they can have more levels of complexity that cannot be subsumed transitively or otherwise, as in “one object is to the right and left of another object” (Halford et al., 1998).

#### 2.3.2. Problem Solving and Transfer

Finding problem solutions by drawing on relational correspondences between representations has been recognized as foundational for higher-order cognitive functions, finding problem solutions (Halford & Andrews, 2006; Gick & Holyoak, 1980), creative innovation (Chan & Schunn, 2015; Dumas, 2018), and knowledge transfer from one context or problem to another (Gentner et al., 1993; Goldwater & Schalk, 2016; Richland & Simms, 2015) across a wide range of academic disciplines, especially including Science, Technology, Engineering, and Mathematics (STEM) (i.e., Dunbar, 1995; Farrington-Flint et al., 2007; Klein et al., 2007; Gentner et al., 2001; Zhao et al., 2021). The key role of relational reasoning in solving problems involves finding relational correspondences between representations and applying it to a new context. The reasoner must construct a mental representation of a problem or a prior exemplar in which they consider both the key features and their relationships (e.g., that one object cuts another into two). Then the reasoner aligns these two representations (e.g., both had one object cut another into two). The objects are alike, as are the “cut” relations (Rattermann & Gentner, 1998). This alignment enables several steps of reasoning: it provides the opportunity for drawing inferences about this new context by allowing one to better understand key features of both the context and the concept, to draw on what happened in one context to determine steps to provide a solution in the second, or to otherwise reorganize one’s mental representation of a new context (see Goldstone et al., 1991). Moreover, core mechanisms of relational reasoning, such as structural alignment and relational abstraction support conceptual understanding and transfer in educational contexts. These mechanisms allow learners to discern relational correspondences across representations, potentially promoting conceptual differentiation by highlighting non-alignable features and, in some cases, prompting conceptual change. Relational abstraction further allows learners to extract schemas that can generalize beyond surface features, thereby laying the foundation for transfer. Consequently, instructional designs that incorporate carefully aligned, multiple comparisons are particularly effective for fostering conceptual change and transfer. In contrast, single, highly concrete analogies may support initial sensemaking but carry a greater risk of misunderstanding and are often less effective for promoting flexible, generalizable understanding (see more discussion in the Limitations and Future Directions Section).

We next turn to elucidating the centrality of relational reasoning to education as an endeavor.

## 3. Learning by and for Relational Reasoning

Relational reasoning as a powerful cognitive tool can contribute to a deeper understanding of key content knowledge in many academic subjects, especially STEM. Beyond its role as a learning strategy, deliberately engaging in relational reasoning—both within and across domains—should itself be an educational objective, because it cultivates expert-like thinking and supports creative problem solving.

### 3.1. Relational Reasoning as a Tool for Learning Domain Knowledge

Since many concepts in STEM or social sciences are inherently relational in nature, students need to attend to and reason about the relations among the concepts to achieve deep understanding. For example, reasoning about relations is fundamental to understanding key mathematical concepts, such as fractions, decimals, and proportions (e.g., Begolli & Richland, 2016; DeWolf et al., 2016). For instance, when learning proportion, students need to focus on the higher-order relation between the two sets of objects and realize that the quantitative relation within each set of objects is the same across them (e.g., it takes 0.6 cups of flour for every strawberry to make a certain flavored cake, regardless of whether it is for a small cake which uses five strawberries and 3 cups of flour or a big cake which uses fifteen strawberries and 9 cups of flour). Relational reasoning plays a similarly important role in science learning. Many key concepts in science are grounded in abstract relations. For example, the concept of force describes and analyzes how two or more entities interact with each other. Being aware of these insights, the Next Generation Science Standards (NGSS Lead States, 2013) aimed to reshape science instruction so that it focused on interconnected systems and relations rather than on discrete facts or topics. Considerable empirical evidence also supports these propositions that relational reasoning ability is closely associated with academic performance in various domains, such as reading (Goswami & Mead, 1992), mathematics (Gentner et al., 2001; Borriello et al., 2023; White, 1998), engineering (Chan & Schunn, 2015; Dumas et al., 2016), and science (Braasch & Goldman, 2010; P. K. Murphy et al., 2017).

### 3.2. Relational Reasoning as an Educational Goal on Its Own

**Figure 1:**
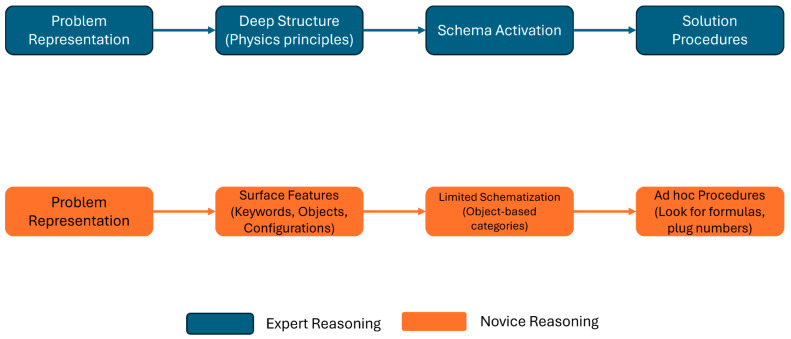
The steps of expert reasoning vs. novice reasoning (Chi et al., 1981).

In addition to serving as a powerful learning technique, relational reasoning is a core educational goal on its own. Expertise in many domains develops through a shift from attending to surface-level features to recognizing and reasoning based on deeper relational patterns (Chi et al., 1981; Goldwater & Gentner, 2015; Rottman et al., 2012). For instance, Chi and colleagues (Chi et al., 1981) examined how experts (i.e., advanced PhD students studying physics or physicists) and novices (i.e., undergraduate students studying physics) categorize and represent physics problems through four experiments involving problem sorting and verbal protocols (Figure 1). In these experiments, participants were asked to categorize or think aloud the approach to solving a series of physics problems. These problems can either be classified by surface-level similarity (e.g., problem format or theme) or structural similarity (e.g., the underlying principles shared across problems despite different problem properties). The findings consistently demonstrated that experts categorize problems based on underlying physics principles or deep relational structures (e.g., Conservation of Energy or Newton’s Second Law) that guide their solution approaches; in contrast, novices categorize the problems based on superficial features mentioned in the problem (e.g., force problem, rotational motion problem). The study highlights that the ability to perceive the deep structure from the surface features is a hallmark of expertise in physics problem-solving.

Goldwater and Gentner (2015) further investigated the processes underlying one aspect of expertise: the ability to detect causal relational patterns across contexts that vary in surface-level characteristics (i.e., common effect, common cause, positive feedback, and causal chain in biology, economics, environmental science, and mechanical engineering). The researchers conducted experiments in which 97 college students learned about specific causal patterns through examples, varying whether students received direct instruction about the causal patterns or engaged in alignment by comparing pairs of examples across contexts. The researchers used an ambiguous sorting task consisting of 16 example phenomena, representing a matrix of the 4 causal systems crossed with the 4 content domains, to measure how participants learned causal system categories when they competed with domain information. The findings indicated that, while directed instruction improved the understanding of individual examples, analogical comparison fostered the abstraction of the common causal pattern. Crucially, the combination of both explication and alignment led to the greatest gains in the sensitivity to the causal patterns in new examples across domains. This finding also aligns with evidence that expert geoscientists spontaneously generate analogies grounded in key causal principles, reinforcing the idea that sensitivity to relational structure characterizes expert cognition (Goldwater et al., 2021). For more discussion of the centrality of relational thought to education in disciplines including science, math, and even history, see Richland and Simms (2015).

In part, the importance of relational reasoning across disciplines pertains to the fact that relational reasoning underlies the development of flexible knowledge transfer and creative problem solving. This is increasingly central to the 21st century educational mission, with national reports proposing that transforming academic content and transferring knowledge across contexts are not only the key to academic competency but also to career opportunities in the 21st century (National Research Council, 2012; U.S. Department of Education, 2023). Persistent difficulties in transferring knowledge to novel situations can often be traced to failures in relational categorization: learners overlook the deep structural commonalities linking superficially different problems (Goldwater & Schalk, 2016). Elevating relational reasoning, therefore, offers a promising remedy. When instruction invites students to refine, manipulate, and interconnect their knowledge instead of treating it as fixed, static, and isolated, they develop a more flexible and well-retained cognitive network that supports effective application (Richland & Simms, 2015).

In addition, relational reasoning also plays an important role in developing and assessing creativity. Creativity is defined as a process in which individuals generate ideas and solutions that are both novel and useful (Dumas et al., 2021; Runco & Jaeger, 2012). The novelty dimension of creativity is represented as the degree to which an idea or solution diverges from conventional ideas or established solutions to a problem, which is sometimes operationalized as semantic distance (Dumas et al., 2021; Kalra & Richland, 2022). Highly creative thoughts or solutions may share fewer perceptual features with conventional ones, reflecting their novelty, but may potentially share the same or similar relational structures, ensuring that they are considered useful. Recent research studies have also investigated and supported the link between creativity and relational reasoning (Chan & Schunn, 2015; Dumas, 2018; Dumas et al., 2021; Green et al., 2012; Vartanian, 2012).

Chan and Schunn (2015) observed a team of 10 professionals from a range of design-related disciplines, such as mechanical engineering, electronics and business development, and industrial design; they were observed in a naturalistic setting when they brainstormed conversations for developing a new product concept for a new market. The researchers examined whether and how relational reasoning, specifically far analogies, led to the generation of creative ideas. Researchers recorded and transcribed two group meetings. Analogy (i.e., near or far) was identified and coded when a designer referred to another source of knowledge and attempted to transfer concepts from that source to the target domain. Near-analogy involved mappings from sources that were related to tools, mechanisms, and processes associated with graphical production and printing, whereas far-analogy referred to mappings from sources which were more distant from the subject at hand. The outcome variable of interest was the novelty of concept generation, which was operationalized as functional distance: the degree to which a given concept’s described functionality was different from a prior concept, rated on a scale ranging from 1 to 5 with 1 being very similar and 5 radically different. They found that far analogies contributed to novel ideas through detailed and focused exploration of the discussed ideas related to the problem, which leads to new and useful versions of the existing concepts in an iterative way.

Dumas (2018) assessed relational reasoning ability and divergent thinking among 77 college students. He used the Test of Relational Reasoning (Alexander et al., 2015), a visual–spatial measure with 32 items in total tapping on four forms of relational reasoning, and the Alternate Uses Task to assess divergent thinking, in which participants were asked to produce as many novel uses for a given object as possible (Guilford, 1967). Two dimensions of divergent thinking were scored—fluency and originality. Fluency was operationalized as the number of uses generated by each participant for each object, and originality was scored as the semantic distance between each participant-generated use (e.g., read; throw like a Frisbee) and the object-prompt (e.g., book) using Latent Semantic Analysis. Quantile regression analysis was then conducted to explore the potentially different predictive effects of relational reasoning ability at varying levels of divergent thinking. The study found that relational reasoning ability significantly predicted originality, a key aspect of divergent thinking, particularly for students scoring at or below the median of originality. Relational reasoning was found to significantly predict fluency only for children scoring at the 90th percentile, implying that the association between relational reasoning and fluency might be confined to those who produce many ideas on the Alternative Uses Task. Therefore, relational reasoning may be an important process or tool in the development of creativity.

Taken together, teaching students how to engage in relational reasoning and abstract relational structures underlying various contexts that differ in surface-level features should be a key educational goal.

## 4. Best Practices for Teaching by Relational Reasoning

Beyond training youth to conceptualize their learning as relational inquiry, designing relational reasoning opportunities for teaching content can have strong implications for teaching practice so that encoded and stored constructs are conceptual, meaningfully embedded in prior knowledge structures, and transferable to new contexts. We review the literature on this topic, highlighting the key ways in which teaching can be supported through analogy or relational reasoning opportunities.

### 4.1. Using Analogy to Fuel Understanding of Abstractions

Analogy is the mental alignment of the systems of relations within two representations, such as between the relationships that govern electric circuits and city water system. For a classic analogy, the higher-order relationship between the two systems of relations (here both “pumped by pressure and circulate”) is “same” (Gentner, 1983). The act of making such analogies allows the reasoner to not only be able to explain the specific representations being elaborated, but also it can fuel an abstraction process in which the reasoner recognizes the common pattern to both representations. This learning mechanism was identified in early problem-solving studies (Gick & Holyoak, 1983), but further research has highlighted how it can be leveraged to teach real-world classroom materials (See Richland & Simms, 2015; Vendetti et al., 2015). In history, for example, a teacher might instruct her class to notice how the U.S. Tea Party rebellion was akin to Native Americans’ attempts to resist U.S. colonization in the Battle of Wounded Knee. Importantly, such analogies not only lead to identification of similarities—where both are actions of a colonized people seeking to separate from the economic and literal domination exerted by another society—but may also highlight key differences. The short- and long-term outcomes in the two battles were strikingly different, with the Tea Party being non-violent, and the Battle of Wounded Knee being a massacre. Further, U.S. colonists ultimately threw off the mantle of the British colonizers, while the Native Americans’ sovereignty was compromised, entering a unique status of protected sovereignty subsumed by the United States government. After reviewing the two examples of colonial resistance, if a student also learned about resistance acts conducted in India and Brazil, she might induce and store an abstraction about colonialism. Perhaps, understanding what colonialism means—one nation conquering another—or drawing the developmental insight that communities who have been led by a conquering nation might be forced to fight back to reclaim their sovereignty. This process of abstraction leads to greater flexibility of that insight or concept (see Gick & Holyoak, 1983), meaning it is more likely to be retrieved when the surface features of the original source and target items are not present in a new target context. If this same student was next to be introduced to the context of South Africa, for example, she might recognize that this is again a colonial context, despite being on a different continent, involving distinct countries and cultures, and engaging different processes of resistance.

Thus, instruction by analogy can lead to several different opportunities for building knowledge. First, these rich comparisons yield stored knowledge representations that are embedded in a network of meaningful higher-order relationships (see Richland & Simms, 2015 for further discussion). By doing so, the likelihood that a learner will be able to recall instructed representations is higher, and the likelihood of recognizing relations to related information is also increased (Gick & Holyoak, 1980, 1983). This can be affected by the effort of the analogy-making. One dimension is the distance of the relationship between the source and target analog. Near analogies typically share some surface features or domain context between source and target: for example, two rate problems in mathematics, with one that uses the context of driving and one that uses the context of bicycling. Far analogies typically do not share surface features and may cross domains, such as between an electric current and water in a garden hose.

#### 4.1.1. Practice: Supported Use of Far-Distance Analogies

Halpern and colleagues (Halpern et al., 1990) demonstrated that the distance of an analogy can impact retention with greater effort leading to greater retention. They found far analogy aided understanding and retrieval of scientific information. In that study, 193 adult participants between the ages of 17 and 64 read three texts on the topics of the lymphatic system, enzymes, and electricity, each with either a near analogy, (e.g., comparing the pull of gravity to electricity flowing through an electrical circuit), a far analogy, (e.g., comparing electric current to water in a garden hose), or no analogy. All combinations of near, far, and no analogy appeared equally often in combination with the three topics. Immediately after reading the text and one week later, participants were assessed on the information retained by four types of tests: (a) a free recall where participants wrote as much as they could remember about each text; (b) a cued recall, in which participants answered the prompted questions (e.g., “Why are metals good conductors of electricity?”); (c) a new analogy item based on the information provided in the text (e.g., “Hydrolysis is to synthesis as scissors is to (1) knife (2) organic solvent (3) acid (4) stapler”); (d) two inference questions for information that could be inferred but not explicitly stated (e.g., “What might happen to the lymph flow if a person were paralyzed?”). The results of a series of ANOVA tests indicated that individuals who read the scientific text with a far analogy outperformed the other two conditions (i.e., scientific text with near analogy or no analogy) on almost all the four tests; that is, they recalled more information in the free recall and cued recall tests, and correctly answered more questions that required inferences or comprehension of a novel analogy both at the immediate and delayed posttests (nonsignificant difference was only found for the solution of new analogy). From an educational perspective, teachers may consider including far analogy examples or practice for students to make efforts in establishing connections across seemingly distant domains for better knowledge retention.

#### 4.1.2. Practice: Teaching by Highlighting Alignable Differences

Such alignable differences have been demonstrated to be particularly salient when all other aspects of the aligned representations share salient features. Kok and colleagues (Kok et al., 2013) demonstrated this in the context of reading X-rays, a notoriously difficult skill due to the many aspects of these visual representations to attend to simultaneously. Though identifying abnormalities within X-rays is challenging, when presented with two X-rays simultaneously that were aligned in all surface features aside from a key difference of physiological importance, participants easily identified discrepancies (see Matlen et al., 2020).

Similarly, when teachers aim to highlight a key difference across representations (e.g., to explain how an underlying mechanism works), learners benefit from providing visual representations that use perceptual similarities to highlight the similarities and alignments. Doing so makes discrepancies especially visible, allowing for the key alignable differences between the representations to be clarified. Hansen and Richland (2020) tested this approach in the instruction of mitosis and meiosis. They provided students with visual representations of mitosis and meiosis, which appeared to be identical and showed the same steps for cellular replication except for illustrating that meiosis includes an additional step in which the cells are split into gametes with half of the total chromosomes. When students saw these two representations lined up on the same page, they were quick to point out the difference between the constructs. This was more effective than having them view and engage with the representations one at a time in sequence, despite the fact that this would likely have reduced their cognitive load to interpret complex diagrams (Mayer, 2014).

Thus, teaching by analogy by providing carefully curated source and target representations to compare can be a potent tool for encouraging abstraction and process learning, in addition to storing and retaining the instructed representations. To use the colonialism analogy begun above, this may lead to improved encoding of both the specific instances that were instructed—here the Boston Tea Party and the Battle of Wounded Knee—and the abstraction that renegotiating colonialist relationships may require uprising. Doing so using representations with similar features but that possess easily aligned relational differences may allow those aspects of the systems that differ in meaningful ways to be noticed.

#### 4.1.3. Explicit Instruction for Students to Use Relational Correspondences

While analogy is potent, it is not the only form of relational comparisons that can be useful with higher-order relations other than “same” being possible across representations. Two relationally complex structures may be causally related, or hierarchically structured, for example. Additionally, the structures may have similar conceptual structure though the details of their representations may not be easily aligned. Crucial to ensuring this is effective for learning, however, is making sure that students do the intended relational reasoning.

A productive and informative study that examined learning from comparisons between mathematical solution strategies demonstrates this instructional tool and opportunities. Mathematics teachers regularly use comparisons to instruct in mathematics (Richland et al., 2006, 2007), perhaps due to the crucial nature of mathematics instruction to teach abstraction, transfer, and flexibility—features that, as described above, are particularly well taught by analogy.

It is more effective to create learning opportunities in which students are required to make comparisons and alignments. In one set of studies, pairs of students were asked to verbally compare and contrast standard and non-standard solution strategies to algebra problems (Rittle-Johnson et al., 2009, 2017; Rittle-Johnson & Star, 2007; Star & Rittle-Johnson, 2008). These opportunities led to gains in procedural knowledge, flexibility, and conceptual knowledge, provided students had sufficient prior knowledge of the key content (Rittle-Johnson et al., 2009; Rittle-Johnson & Star, 2007; Star & Rittle-Johnson, 2008). For example, Rittle-Johnson and Star (2007) randomly assigned seventy seventh-graders to two conditions. In the comparison condition (*n* = 18 pairs), students studied sets of two strategies for solving the same algebraic equation side-by-side in worked problems and discussed their differences. The other condition was the sequential condition (*n* = 17 pairs), where students studied the same two strategies but in two different worked problems presented sequentially, and then they were asked to reflect on one single problem.

Students worked in pairs over two days, solving practice problems and writing explanations. Results of a series of multilevel modeling analyses showed that students in the comparison group made significantly greater gains in procedural knowledge and flexibility, including increased use of more efficient, non-conventional methods. Therefore, comparisons that highlight the structural differences can be a powerful instructional tool to enhance learning.

#### 4.1.4. Generating Explanations and Self-Explanations

Generating explanations and self-explanations are another paradigm that leads to relational construction (Atkinson et al., 2000; Chi et al., 1994; Lombrozo, 2006). Explanations are inherently causal, meaning that children who are asked to explain their thinking, either explaining a process or phenomenon to others, or to themselves via self-explanation, are typically engaging in causal construction of their knowledge, which can develop relational stored representation and lead to both conceptual knowledge and transfer. During the explanation process, children have an opportunity to integrate new material with prior belief, identify gaps in knowledge and update it to accommodate new information, and seek broader and unifying generalizations available for transfer in order to understand the new information that they are trying to explain (see Lombrozo, 2012 for a review). Chi and colleagues (Chi et al., 1994) developed the paradigm of self-explanation, assessing the effect of self-explanation on learning among 24 eighth-grade students. As an assessment of prior knowledge, all students were asked a set of questions about each of the 23 terms on human circulatory system and to draw the path of blood flow throughout the body before the experiment started. Fourteen participants were then asked to read a 101-sentence passage that introduced how the circulatory system works in human beings. After reading each sentence which was printed on a separate piece of paper, these participants were prompted to explain what each sentence means. In addition to these general prompts, they were also prompted to explain the function of 22 key components of the circulatory system, such as atrium. Another ten participants in the control condition were not prompted for either self-explanations or functions. Instead, they were asked to read the same passage twice, which took approximately the same time on average as the total time that participants in the experimental condition took. All students were then asked to answer the same set of questions about the 23 terms on human circulatory system and redraw the path of blood flow. Results showed that the prompted group had a greater gain from the pretest to the posttest compared to the control group. Moreover, students who generated a large number of self-explanations in the prompted group (*n* = 4; high explainers) had a better understanding than low explainers (*n* = 4). Additionally, researchers inferred and drew the mental model of circulatory system for each participant based on their answers and blood path drawing. They found that all the high explainers achieved the correct mental model, whereas many of the unprompted students and low explainers did not.

A large body of research has built on the self-explanation effect paradigm, with much of the work demonstrating that explanations can lead to relational alignment and structure mapping, ultimately leading to learning and abstraction (Lombrozo, 2012).

### 4.2. Challenges and Practices in Relational Instruction

While relational reasoning is a potent and powerful instructional practice, it is also effortful, and there are challenges that must be considered to ensure its efficacy. We describe key challenges that must be considered, with specific practices identified for meeting these needs on the part of students.

#### 4.2.1. Noticing the Utility of Reasoning Relationally

Reasoning relationally often involves disattending to perceptual information and recognizing correspondences between representations that do not share perceptual features. Expertise in a domain leads reasoners to be more likely to notice and focus on relational correspondences (Chi et al., 1981); however, by nature, students are non-experts in the domains in which they are being instructed, so teachers may need to be very explicit about when students should be seeking relational correspondences and when they should be making relational inferences. Researchers have identified several different types of educational practices that can be effective at supporting students in doing so. We describe some of these practices in turn, which include explicit cuing and structuring a learning context to set up progressive alignment.

##### Explicit Cuing

Providing explicit cues for learners to compare prior instructed problems with the target problems improved the likelihood that learners noticed the relations on the structural level between the two types of problems (Blessing & Ross, 1996; Gick & Holyoak, 1980; Novick, 1988; Novick & Holyoak, 1991). Those cues could be general reminding statements for comparison (e.g., “To figure out how to solve Problem 2, think about how we solved Problem 1”) or specific information about the elements that can be mapped productively across problems. For example, when teaching two math word problems, one about baking a cake and one about making lemonade, a teacher might ask: “How are the eggs in the cake like the lemons in the lemonade?”. The role of cues was studied in early experiments to determine whether learners’ difficulty was in encoding and representing relations for transfer or recognizing the need to align representations. Gick and Holyoak (1980, 1983) demonstrated that when explicitly cued, most participants who read two problems were able to map the correspondences and learn from the relationships, so the challenge was in noticing the key relations.

##### Progressive Alignment

Moreover, guiding students through a series of comparisons using a strategy in which one aligns progressive alignment strategy can also draw students’ attention to the structural similarity between two seemingly different types of problems (Kotovsky & Gentner, 1996). The key to the progressive alignment strategy is to start from problem sets that share more surface-level similarities as well as structural similarity, and then gradually move to problem sets that share less and less similarities on the surface level, and finally compare problem sets that only share structural similarity. For example, when teaching the multiplicative relation, y = 1.5x, following the progressive alignment strategy, teacher could first use two recipe problems for students to compare, in which the quantity of one ingredient (i.e., flour/water) is always 1.5 times of the other one (i.e., milk/orange). Then, teacher could use one recipe problem and one painting problem with different numbers from the first pair, in which the quantity of one thing (i.e., flour/green paint) is always 1.5 times of the other (i.e., milk/red paint) in each problem. Finally, the teacher could introduce the equation y = 1.5x and encourage students to map x and y to their correspondences based on the role they play in each of these stories, thus allowing students to understand the multiplicative thinking underlying these problems.

#### 4.2.2. Eliciting a Relational Mindset

While explicit directions to attend to relational correspondences can be an effective practice, it is also important and useful for reasoners to develop an implicit focus on relations. This can derive from an ideological understanding of what constitutes knowledge in a domain, such as creating an understanding of mathematics, science, or history that recognizes the importance of finding the causal and relational structure embedded in the content being taught.

It can also derive from a generalized attention to relationships, in which a growing body of the literature suggests that it may be an individual difference that is relatively stable, or at least a trainable attentional pattern among both adults and children. Currently, researchers have been using the spontaneous scene analogy task to measure this attentional tendency to relations (see A. N. Murphy et al., 2021, for test details). During the task, participants were presented with two pictures depicting relation(s) that were the same but using different objects. Participants were instructed to “choose one of the things in the bottom picture that goes with the xxx in the top picture”. They could match objects across the two pictures either based on relational similarity (i.e., the object that plays the same role as the target from a relational perspective despite looking differently) or based on object similarity (i.e., the object that looks exactly the same despite playing a different role from a relational perspective). Relational attention was measured as the degree to which participants overcame the tendency to attend to object similarity (i.e., selecting the object match), and instead spontaneously attended to the relational similarity by selecting a relational match. The picture materials controlled for number of items per scene, and analyses controlled for executive function and age—related variables that could also explain changes in children’s relational thinking (Zhao et al., 2025a). Vendetti and colleagues (Vendetti et al., 2014) and Walker and colleagues (Walker et al., 2018) demonstrated that adults could be primed to notice relational correspondences by doing a challenging, high-demand relational task—generating solutions to far-distance analogies—before mapping correspondences on this spontaneous scene analogy task. In a developmental study building on this paradigm, Simms and Richland (2019) found that, like adults, young 3–4 year old children who were prompted to generate and articulate the relations between objects in a relational task by themselves were more attentive to relational similarity and less to object similarity in a subsequent task than those who passively listened to an experimenter describing the same relationships.

Moreover, increasing research also suggests that eliciting this spontaneous relational attention or relational mindset may have implications for learning and information processing. Zhao and colleagues (Zhao et al., 2025a) found empirical evidence showing that a relational mindset may have direct benefits in classroom math learning. They used a latent class analysis to explore individual differences in relational attention among 272 elementary school students. Four distinct clusters were identified: (a) Relational Attenders, who consistently attended to relational similarities; (b) Emerging Relational Attenders, who attended to relational similarities but not as consistently as the Relational Attenders; (c) Object Matchers, who consistently attended to object similarities; and (d) Inconsistent Responders, who had an inconsistent pattern in attending to similarities. The researchers then fit a mixture model to explore the potential implications of these individual differences in relational attention on students’ math learning outcomes. They found that children who consistently and spontaneously attended to role-based relations in a non-mathematical relation task (i.e., Relational Attenders and Emerging Relational Attenders) had better learning outcomes from the same 20 min math lesson than their peers who consistently and spontaneously focused on object features (i.e., Object Matchers), controlling for executive functions and prior math knowledge. Therefore, having an implicit attentional pattern that prioritizes relations, may be a powerful tool for achieving optimal learning.

Relatedly, Goldwater and Jamrozik (2019) found that relational mindset could boost retrieving analogical relations from multiple texts read by undergraduate students as long as such relational mindset was elicited before the information was encoded. Following the paradigm of Vendetti et al. (2014), undergraduate participants completed either an analogy task that aimed to elicit their relational mindset or a semantic word-association task serving as a control. They then read multiple texts from different domains, each about a particular relational concept (e.g., “trade-off”, “pre-emption”) explored in a particular domain (e.g., medicine, computer science). Following that, they read another similar set of texts, each could be matched with an article in the previous set based on relation (e.g., both about “trade-off” but in different domains) and domain (e.g., both in medicine but about different relational concepts). The researchers then assessed the degree to which undergraduates retrieved previously read information based on relation (e.g., they recalled a previously read text on “trade-off” in medicine while reading the text on “trade-off” in computer science) or domain (e.g., they recalled a previously read text in computer science but about “pre-emption” instead). They found undergraduate students who completed the analogy task prior to reading the first set of texts retrieved more information based on relations than their peers in the control condition whose attention was not primed to relation before reading, suggesting that high-quality relational encoding was very important for subsequent relational retrieval and that inducing a relational mindset could improve those encodings.

Therefore, it is reasonable to speculate that eliciting a relational mindset before learning activities take place might have implications for the quality of the key information encoded during the learning phase, thus having consequences for learning outcomes. Training young children with a warm-up activity that requires them to actively think relationally is likely to elicit a relational mindset, which might contribute to learning from the subsequent instructions. Zhao and colleagues (Zhao et al., 2025b) demonstrated that a warm-up activity that required children to actively think relationally in a non-explicitly math context successfully directed their attention to mathematical relations in a subsequent, different task. The elicitation of relational mindset also had potential in improving children’s learning from a video math lesson.

#### 4.2.3. Exposure to and Use of Relational Language

Exposure to and using the language of relations can support children in building relational reasoning skills, both through encouraging a relational mindset, as well as to support growth in relational skills over time. This has been examined both in naturally occurring everyday speech, as well as in experimental designs manipulating specific language production. We briefly review these two bodies of literature in turn.

In everyday speech, linguistic and discursive patterns can invite and invoke relational thinking in both the speaker and the listener. Close analyses of everyday home language experiences for U.S. children have revealed discursive patterns of comparison (Silvey et al., 2023) and, more broadly, relational Higher-Order Thinking Talk (HOTT, Frausel et al., 2022). HOTT involves language that is involved in representing, inquiring about, and conceptualizing the world relationally through linguistic practices. We define HOTT specifically to include discourse that links ideas to make inferences, draw comparisons and analogies, construct hierarchies and categories, invoke schemas and link definitions to abstractions. Consider a teacher who asks the following question: “Why do you think this problem ended with *x* = *x*, while that problem ended in *x* = 2? What does that mean about what numbers can be used to satisfy the equations?” This mathematical prompt by a Chinese teacher (Williams et al., 2009) not only draws students’ attention to a distinction that provides meaningful insight into the nature of variables and the equality relationship, but also it socializes students to know that mathematics is a field that requires reasoning to question why patterns emerge.

Patterns of acquisition and usage, as well as the longer-term implications of early HOTT, were investigated in a sample of 60 families selected to reflect the demographic range of Chicago (31 girls; 34 first born children). Spontaneous home talk was videotaped while a primary caregiver was present for 90 min at a time, three times a year, from 14 months through 58 months. All of these interactions were then transcribed systematically, with high reliability calculated between transcribers (see Rowe & Goldin-Meadow, 2009). Each utterance was coded and given a score for whether it displayed or encouraged HOTT, yielding over 1 million utterances coded for analysis.

Crucially, these early HOT utterances, both child and parent, were impactful in predicting future STEM and reasoning skills. Parent HOTT was correlated with SES and parent education level, which were correlated with children’s HOTT onset of usage and frequency (Frausel et al., 2020). Further, children’s early frequency of using HOTT that was structurally meaningful predicted children’s 5th grade performance on a verbal analogies task, in addition to reasoning and inferencing on a reading comprehension task. Thus, these patterns of talk in early childhood may shape the skills that children bring to learning and to educational contexts going forward.

Analyses of these HOTT data also revealed that HOTT was invoked regularly while parents or children told personal narratives—stories about one’s experiences in the past or expected experiences in the future (e.g., a planned trip to the zoo; Frausel et al., 2021). This finding suggests personal narratives may be a leverage point for increasing the amount of HOTT children are exposed to.

Relational language has also been investigated as a tool for improving reasoning on specific tasks (see Loewenstein & Gentner, 2005; Pruden et al., 2011). Loewenstein and Gentner (2005) conducted a series of experiments and found that hearing the language of spatial relations (i.e., “on”, “in”, “under”, “top”, “middle”, “bottom”) enhanced the spatial relational encoding, thus improving young children’s spatial mapping performance. Young children who were provided with the spatial relational language during the mapping or orientation phase from the experimenter, for example, “I’m putting the winner *on/in/under* or *at the top/middle/bottom of* the box”, outperformed children in the baseline condition on a series of spatial correspondence tasks, where such spatial relational language was replaced by general language (e.g., “I am putting the winner *here/there*”) and accompanying gestures, after controlling for age and the location of the target object. The results were consistent across five experiments, and researchers found that the effects of such spatial language were maintained over time. Children who were provided with the spatial terms during the initial orientation phase outperformed their peers in the baseline condition when both groups carried out the mapping task two days later, with no further exposure to the spatial terms.

In another longitudinal study the same interactions that had been coded for HOTT (Frausel et al., 2021) were now coded for a specific type of relational language—spatial language (Pruden et al., 2011). In the dataset used for this analysis, researchers examined the frequency of spatial language inputs in data collected with 52 children between the ages of 14 months and 46 months in a total of nine visits, in which unstructured, spontaneous home interaction was recorded once every four months for 90 min. In addition to these naturalistic observations, researchers also evaluated children’s performance on three non-verbal spatial tasks and their receptive vocabulary when they were at 54 months of age. When analyzing the video transcribed data, researchers focused on the use of language about the spatial properties and features (e.g., “circle”, “triangle”, “big”, “little”, “bent”, “curvy”) from parents and children separately. Considerable individual differences in the use of spatial terms were found for children and parents (i.e., 5 tokens of spatial language to 525 spatial tokens for parents, 4 to 191 spatial tokens for children over the 9 visits). Importantly, the study found that, when parents tend to use more spatial language, their child was likely to use more spatial language, and vice versa (*r* = 0.55, *p* < 0.001), after controlling for parent’s overall language use. Moreover, children who produced more spatial language were also likely to perform better on spatial problem-solving tasks at a later age (0.33 ≤ *r*s ≤ 0.39, *p* < 0.05) after controlling for the parent’s and the child’s overall language use.

#### 4.2.4. Activating Relevant Prior Knowledge

Adequate prior knowledge is necessary to facilitate and enable productive relational reasoning. The developmental literature on analogy originally derived from Piaget’s assertion that children were not able to reliably solve analogy problems until they had reached formal operations, typically adolescence, due to their low success on his analogy problems until that point (see Goswami, 2002 for discussion). New paradigms that took children’s knowledge base into account and ensured children had strong understanding of all included relations demonstrated, in contrast, that children could reason reliably about relations by 3–4 years of age (see Goswami & Mead, 1992; Rattermann & Gentner, 1998). Therefore, adequate prior knowledge is necessary for children to engage in relational thinking, though it can be a mechanism of learning as well.

Importantly, Gentner (1988) posited the relational shift, which is the observation that, as people build knowledge in a domain, they move from noticing and reasoning on the basis of perceptual features to noticing and reasoning on the basis of relational structure. For example, this was observed in adults in a business school learning context. When asked to compare cases, students first matched by finding alignments between the surface content of the cases, while with greater knowledge, they mapped on the basis of relations (Loewenstein & Gentner, 2005).

Children also need sufficient prior knowledge to benefit from analogical comparisons. In an algebra task in which students were assigned partners and randomly assigned to conditions in which they were asked to either compare solution strategies simultaneously or to discuss them one at a time in a sequence, students’ level of algebra knowledge at the introduction to the lesson was predictive. If on a pre-test the students made any attempt to solve algebra problems by moving calculations across the equal sign, showing some level of prior knowledge in solving equations, even if incorrectly, they were likely to learn better from the comparison condition than the sequential condition. When students did not begin the intervention with these early skills; however, they benefited more from learning about the problem solutions separately (Rittle-Johnson et al., 2009). Therefore, ensuring and potentially activating the relevant prior knowledge allows learners to engage in and benefit from relational instruction.

#### 4.2.5. Alleviating Cognitive Load to Support Relational Reasoning

Relational reasoning relies heavily on executive functions, required for mentally representing relational representations, aligning and mapping, inhibiting irrelevant correspondences, and inferring structure and information about problem solutions (Krawczyk, 2012; Krawczyk et al., 2008; Morrison et al., 2004; Waltz et al., 1999). Considerable evidence from neuroscience supports the link between prefrontal cortex—an area that primarily governs including selective attention, inhibitory control, and working memory—and performance on relational reasoning tasks. Krawczyk and colleagues (Krawczyk et al., 2008) and Morrison and colleagues (Morrison et al., 2004) investigated adults with frontotemporal lobar degeneration (FTLD) and found that those with damage to the prefrontal cortex performed more poorly on relational reasoning tasks than those with damage to the temporal lobe, particularly when the tasks demanded greater inhibition of distracting information. Similarly, children with traumatic brain injury may display lower levels of relational reasoning, in part explained by damage to the executive function resources (Krawczyk et al., 2010). In young children, EF resources that are still limited by development means that children are more susceptible to inhibition failures (see Richland et al., 2006). Researchers found that children who were successful at mapping relations in scene analogies performed at chance when required to inhibit responding to a perceptually salient lure.

Educationally, these mechanisms have implications for how one might structure a pedagogical context to reduce the burden on learners, inhibiting attention to irrelevant correspondences, and to align and map key relational correspondences. The specific details of how one presents information during instruction may be crucial to predicting whether learners will notice key relations, represent the information relationally in mind, and align and map relevant and crucial relational structures. Doing so in a way that reduces the cognitive load for conducting structural mapping can improve the likelihood that this is the case (Catrambone & Holyoak, 1989; Gick & Holyoak, 1983; Novick & Holyoak, 1991; Richland & McDonough, 2010; Rittle-Johnson & Star, 2007; Ross & Kennedy, 1990; Star & Rittle-Johnson, 2008). Some of the these commonly used instructional approaches include presenting the source analogs (e.g., two problems or strategies) simultaneously and making them visible together while they are being instructed (see Begolli & Richland, 2016; Kok et al., 2013), using visual alignment and linking gestures to highlight to correspondences between items being compared, and encouraging mental imagery or visualizations for comparisons (Alfieri et al., 2013; Matlen et al., 2020; Richland & McDonough, 2010).

##### Reducing Cognitive Load by Making Comparisons Visible: Simultaneous Versus Sequential Presentation of Representations

Viewing two representations simultaneously can result in high cognitive load due to requiring a large amount of information to organize attention to find and map relational correspondences (Mayer, 2014). Richland and McDonough (2010) conducted two experiments to assess the degree to which these instructional approaches that should facilitate structural alignment by reducing cognitive load would enhance learning and transfer. College participants learned mathematical concepts (e.g., permutation, combination) through carefully controlled video instructions that varied in the level of cuing to relational structure, including comparative gesture, source visually supported, source visible during instruction of target, visual alignment, and the opportunity for students to actively engage in judging the accuracy and mapping correspondences between different strategies. They found that the high-cuing condition resulted in higher rates of positive extension of learning to new contexts, and lower rates of susceptibility to misleading contextual features when assessed both immediately and one week later.

Further work since that laboratory-based study has examined the benefits of these practices in more ecologically valid educational contexts, including in classroom mathematics lessons on proportional reasoning (see Begolli et al., 2018 for review), as well as in science. As described above (Hansen & Richland, 2020), providing children with opportunities to learn from meiosis and mitosis representations on the same page was more effective for learning than learning from them in sequence.

In another quite different mode of instruction, in a game-based math learning paradigm, similar results were identified for teaching rational number conversion using opportunities for relational reasoning (Bustamante et al., 2022). When compared to business as usual, games that required students to compare representations and move between decimal and rational number representations led to greater learning and transfer to new rational numbers. For those who began with adequate skill, a test of simultaneous versus sequential instruction also showed similar results to those described above. Games either incorporated multiple different denominators that had to be converted to decimals on the same game play board, or they were kept separate onto different game boards. When taught together, the overall learning was higher, and there was better transfer to new number representations for the simultaneous representations, but this did depend on students’ prior knowledge.

These results provide indication that these cognitive principles of analogy and relational reasoning can inform instruction productively through traditional instruction as well as more innovative approaches such as game play.

To this end, recent developments in large language models (LLMs) and AI have also led to exciting opportunities and possibilities. While generative AI does not seem to process higher-order relations using similar representational approaches to a human, there has been rapid evidence for the emergence of relational reasoning capabilities in these systems. This indicates this may be a productive way to support relational reasoning in academic contexts in youth and learners as well, since engaging learners in HOTT or relational reasoning requires the skill to participate in this level of thought as well. Webb and colleagues (Webb et al., 2023) demonstrated that models such as GPT-3 and GPT-4 can perform at, and in some cases even above, human levels on a variety of analogical tasks, including problems structurally similar to Raven’s Standard Progressive Matrices. Lu et al. (2019) also developed computational models capable of generating analogical mappings from initially nonrelational inputs. Theoretically these advances suggest there are different pathways for conducting analogical reasoning, since LLMs may not perform relational representation as a human might, but most importantly for the current manuscript, this work indicates the potential for computational systems to generate and develop analogical reasoning opportunities. These developments suggest that AI has the potential to become a valuable aid for relational instruction. For example, teachers might use AI to generate problem sets that differ in surface features but share deep structural similarities, allowing students to compare them. Similarly, students could engage AI tools to explain analogical mappings step by step, deepening their understanding of the underlying relations.

## 5. Limitations and Future Directions

Overall, the deep cognitive literature on relational reasoning has great potential for guiding and contributing to instructional practice in several key educational domains, yielding precisely the type of flexible, generalizable learning that is increasingly important to the demands of the 21st century.

Although we have emphasized the utility and benefits of engaging in relational reasoning for learning, it is also important to acknowledge its limitation. In particular, analogical reasoning can sometimes mislead learners away from the intended learning goals to forming misconceptions, especially when learning complex concepts. As highlighted by Spiro and colleagues (Spiro et al., 1988), exact one-to-one mappings between source and target domains are rare (i.e., all the key relations in the source can be mapped one-on-one to all key relations in the target); more often, only a subset of key elements in the target aligns with the source. While such partial mappings may help novices gain an initial understanding of the target, they risk producing incomplete or even erroneous conceptions. Misunderstanding can arise when the source domain of an analogy is inadequate or potentially misleading for understanding the target domain. In such cases, learner’s understanding may be reduced to only those features explicitly mapped from the source, leading to oversimplified or distorted representations of the target concept. For example, when household plumbing is used as an analogy for teaching blood flow in human body, learners may focus on resistance as the sole cause of impedance in blood flow while overlooking critical nonaligned properties, such as the flexibility of blood vessels in contrast to the rigidity of pipes. As a result, students may struggle to understand or may entirely neglect additional sources of impedance in blook flow, such as compliant and inertial reactance, which play essential roles alongside resistance. This tendency to reduce complex systems to the core relations highlighted by a single analogy exemplifies how analogical instruction, if not carefully scaffolded, can foster entrenched misconceptions. Moreover, these oversimplified representations can be resistant to change, persisting even as learners progress to more advanced stages where a deeper understanding of the target’s complexity is required. In addition, teaching by analogy runs the risk that young learners may focus on surface features or irrelevant relations when mapping across domains. Zook and Di Vesta (1991) similarly found that children who learned through analogy generated more incorrect inferences from surface-level similarities or irrelevant features than those who were not made aware of the analogy, consistent with the relational shift hypothesis.

Thus, when using analogy in instruction, particularly with young learners, teachers should anticipate where misalignments may occur and proactively mitigate them through explicit guidance.

## 6. Discussion and Implications

This study therefore indicates a set of applications for educational practice that are both important directions for further research, bridging the cognitive science of relational reasoning and education, and that are usable principles for informing practice. We summarize these here as an opportunity to consider direct ways to use this research for instruction and encourage researchers to test their relevance across disciplinary foci. Though teachers who are not attuned to the use of comparison may not adopt these practices easily (see Star et al., 2015), training that incorporates teacher professional development along with curriculum materials designed to clarify meaningful comparison and contrast opportunities may be a productive way to support adoption of these practices (see Durkin et al., 2023).

1. ***Ensure a learner recognizes the utility of relational reasoning:*** This may take the form of directed attention with speech that highlights the usefulness of referencing a prior referent, use of relational language, or drawing attention to one-on-one relational correspondences within representations. This can also take the form of using spatial or imagery cues to highlight these correspondences, such as making two representations visible and aligned spatially. The implementation will vary across disciplinary content due to the nature of disciplinary knowledge. In mathematics, for example, a teacher might explicitly highlight the relevance of multiple solution strategies or problems. The teacher might explicitly state a cue such as the following: “Solve this problem the same way as the last one”. Alternatively, the teacher might use spatial cuing in which two problems are written on the board with their solution steps spatially aligned (see (Richland et al., 2007) for more examples from classroom practices). In science, this might include a teacher displaying a model and cuing the students into how to align this with the real-word scientific system (system of relationships). In history, this could look different, with cuing for example taking the form of asking students to develop an outline of key events or patterns of relationships such political systems and draw connections between similar processes across nation-states.
2. ***Build on relevant prior knowledge:*** Ensuring that reasoners have a strong knowledge base for conducting analogical reasoning is important, as this is a prerequisite for successful structure mapping and inferencing. Analogy may support building knowledge but relies upon adequate understanding of the representational structures to be used successfully. The timing may matter, such that opportunities for relational reasoning may support building schemas and more flexible transfer, but without adequate prior knowledge, students will need additional support to make these effective. Thus, we recommend that teachers check or enhance students’ understanding of the key concepts they have acquired that will be needed to engage in relational reasoning when learning the new content. For example, when teaching proportions in a math class, before introducing proportions, teachers could begin with a few review questions of fraction, a key concept for learning proportions.
3. ***Support novices in a field in developing a relational mindset***: Expertise in many domains is evidenced through a focus on relations within the field (Chi et al., 1981; see Richland & Simms, 2015); thus, novices may build their expertise through learning that is organized relationally. Instructionally, this can be supported by using relational language and learning prompts that highlight the importance of retaining relational versus object-based information or asking students to generate explanations that invite relational and causal thinking. For example, when teaching proportions, teachers could direct students’ attention to the relation between lemon and water in two lemonades of different sizes and invite students to explain why the flavor of two lemonades stays the same despite different sizes. For young children, supporting domain-general opportunities to socialize attention to relations may be useful in fostering a relational mindset. That means children may bring those attentional patterns into new domain learning contexts, thereby supporting domain-specific knowledge building that is more expert-like and relational. This can be facilitated by socialization practices including asking open ended questions, using relational language, and setting up opportunities for children to notice relations or use relational language.
4. ***Use and Support Learners in Using Relational Language***: Using relational language can direct attention in a particular learning opportunity to key relational correspondences, or to a mode of thinking that invites relational reasoning. Relational language may include specific cues that highlight the relevant relation such as “above” or “halved.” They may also be deictic terms that draw attention to correspondences across relational structures: “This one is like that one” (Alibali et al., 2014; Richland & Simms, 2015). More broadly, Higher-Order Thinking Talk (HOTT) can be used in many home or school contexts across tasks or domain contexts in order to shape children’s relational skill development, their mindsets, and their school learning. These are relational words such as those listed above, as well as categorization, generalization, and related language processing. Most important is to set up a learning context to scaffold learners to use relational language themselves, which is more impactful than having the knowledgeable instructor to generate that language. Going back to the example of learning proportions, teachers could structure the lesson using comparison pedagogy, such as comparing different proportional problems with different cover stories and/or comparing different solution strategies for the same proportional problem. More importantly, teachers should have a review session intended for students to carry out the comparisons and their own explanations, which would elicit relational language and HOTT.

Altogether, these instructional practices are all feasible, low-resource strategies for encouraging learners to reason relationally while learning traditional academic subjects, leading to both transferable knowledge and a more expert-like approach to understanding domain knowledge. These practices encourage noticing the relevance of aligning and mapping relations, training students to look for these opportunities, or approaches to helping students who are by definition novices in a domain disattend to surface features and instead prioritize structural/more conceptual correspondences.

## Data Availability

No new data were created or analyzed in this study. Data sharing is not applicable to this article.

## Note

1 The studies reviewed in this article were selected for their relevance to the major themes of the manuscript and the key theories in the field of relational reasoning, as well as its subfields. These subfields include relational category acquisition, the role of relational reasoning in other domain-general skills (e.g., problem solving, knowledge transfer) and domain-specific knowledge (e.g., mathematics), spontaneous relational attention, and evidence-based teaching practices that support relational reasoning. We selected classic theoretical work, such as Dedre Gentner’s structure mapping theory, foundational empirical studies, and more recent empirical work on spontaneous relational attention and large language models. The core set of articles was identified primarily through the authors’ expertise in the field and hand-searching publications by prominent researchers in the field. This set was then expanded through snowballing methods by reviewing the reference lists of these key articles. Please see a complete list of studies included in this review in the Appendix A.

## Appendix A

**Topics**	**Included Articles**
Significance of relational reasoning in the broad educational context	Darling-Hammond, L. (2022). Reimagining American education: Possible futures: The policy changes we need to get there. *Phi Delta Kappan*, *103*(8), 54–57. https://doi.org/10.1177/00317217221100012 Darling-Hammond, L., Alexander, M., & Hernández, L. E. (2024). *Redesigning high schools: 10 features for success.* Learning Policy Institute. National Research Council. (2012). *A framework for K–12 science education.* National Academies Press. NGSS Lead States. (2013). *Next generation science standards: For states, by states.* National Academies Press. OECD. (2025). *Trends shaping education 2025.* OECD Publishing. Richland, L. E., & Simms, N. (2015). Analogy, higher-order thinking, and education. *Wiley Interdisciplinary Reviews: Cognitive Science*, *6*(2), 177–192. U.S. Department of Education, Office of Educational Technology. (2023). *Artificial intelligence and the future of teaching and learning.* World Economic Forum. (2023). *Defining education 4.0: A taxonomy for the future of learning.*
Theories of relational reasoning/higher-order thinking	Alexander, P. A., Dumas, D., Grossnickle, E. M., List, A., & Firetto, C. M. (2015). Measuring relational reasoning. *Journal of Experimental Education*, *84*, 119–151. Alexander, P. A., & The Disciplined Reading and Learning Research Laboratory. (2012). Reading into the future. *Educational Psychologist*, *47*(4), 259–280. Bloom, B. S. (1956). *Taxonomy of Educational Objectives: The Classification of Educational Goals; Handbook I: Cognitive Domain.* New York: Longman. Dumas, D., Alexander, P. A., & Grossnickle, E. M. (2013). Relational reasoning in educational contexts. *Educational Psychology Review*, *25*, 391–427. Gentner, D. (1983). Structure-mapping: A theoretical framework for analogy. *Cognitive Science, 7*, 155–170. Gentner, D. (2003). Why we’re so smart. In *Language in mind* (pp. 195–235). Gentner, D., Holyoak, K. J., & Kokinov, B. (Eds.). (2001). *The analogical mind.* MIT Press. Goldstone, R. L., Medin, D. L., & Gentner, D. (1991). Relational similarity. *Cognitive Psychology*, *23*, 222–262. Holyoak, K.J., Thagard, P. (1989). Analogical mapping by constraint satisfaction. *Cognitive Science*, *13*, 295. Lewis, A., & Smith, D. (1993). Defining higher-order thinking. *Theory Into Practice*, *32*(3), 131–137. Resnick, L. B. (1987). *Education and learning to think.* National Academy Press. Spellman, B. A., & Holyoak, K. J. (1992). If Saddam is Hitler then who is George Bush? *Journal of Personality and Social Psychology*, *62*, 913–933.
Relational category	Doumas, L. A., & Hummel, J. E. (2013). Comparison and mapping facilitate relation discovery. *PLoS ONE*, *8*, e63889. Goldwater, M. B., & Schalk, L. (2016). Relational categories as a bridge. *Psychological Bulletin, 142*, 729–757. Kalra, P. B., & Richland, L. E. (2022). Relational reasoning. *Mind, Brain, and Education*, *16*, 149–152.
Relational reasoning in problem-solving, creativity, and transfer	Catrambone, R., & Holyoak, K. J. (1989). Contextual limitations on transfer. *JEP:LMC*, *15*, 1147–1156. Dumas, D. (2018). Relational reasoning and divergent thinking. *Contemporary Educational Psychology*, *53*, 1–14. Dumas, D., Dong, Y., & Doherty, M. (2021). Creative expertise and analogical reasoning. *Mind, Brain, and Education*, *15*, 239–249. Dumas, D., Schmidt, L. C., & Alexander, P. A. (2016). Predicting creative problem solving. *Thinking Skills and Creativity*, *21*, 50–66. Dunbar, K. (1997). How scientists think. In *Conceptual structures and processes*. Gentner, D., Rattermann, M. J., & Forbus, K. D. (1993). Similarity in transfer. *Cognitive Psychology*, *25*, 524–575. Gick, M. L., & Holyoak, K. J. (1980). Analogical problem solving. *Cognitive Psychology*, *12*, 306–355. Gick, M. L., & Holyoak, K. J. (1983). Schema induction and analogical transfer. *Cognitive Psychology*, *15*, 1–38. Green, A. E. et al. (2012). Neural correlates of creativity in analogical reasoning. *JEP:LMC*, *38*, 264. Guilford, J. P. (1967b). *The nature of human intelligence*. McGraw-Hill. Halford, G. S., & Andrews, G. (2006). *Reasoning and problem solving.* Handbook of child psychology, Volume 2, Cognition, perception, and language. D. Kuhn, R.S. Siegler, W. Damon & R.M. Lerner (Eds). New Jersey USA: John Wiley & Sons, Inc. 557–608. https://doi.org/10.1002/9780470147658.chpsy0213 Kalra, P. B., & Richland, L. E. (2022). Relational reasoning: A foundation for higher cognition based on abstraction. *Mind, Brain, and Education*, *16*(2), 149–152. Loewenstein, J., & Gentner, D. (2005). Relational language. *Cognitive Psychology*, *50*, 315–353. Markman, A. B., & Wood, K. L. (Eds.). (2009). *Tools for innovation.* Oxford University Press. Novick, L. R. (1988). Analogical transfer and expertise. *JEP:LMC*, *14*, 510. Richland, L. E., & Simms, N. (2015). Analogy, higher-order thinking, and education. *Wiley Interdisciplinary Reviews: Cognitive Science*, *6*(2), 177–192. Ross, B. H., & Kennedy, P. T. (1990). Generalizing from earlier examples. *JEP:LMC*, *16*, 42–55. Runco, M. A., & Jaeger, G. J. (2012). The standard definition of creativity. *Creativity Research Journal*, *24*, 92–96. Skemp, R. R. (1979). *Intelligence, learning, and action.* Wiley. Vartanian, O. (2012). Neural systems for analogy and metaphor. *British Journal of Psychology*, *103*, 302–316.
Relational reasoning in domain-specific knowledge acquisition	Alfieri, L., Nokes-Malach, T. J., & Schunn, C. D. (2013). Learning through case comparisons: A meta-analytic review. *Educational Psychologist*, *48*, 87–113. Begolli, K. N., & Richland, L. E. (2016). Teaching mathematics by comparison: Analog visibility as a double-edged sword. *Journal of Educational Psychology*, *108*(2), 194–210. Borriello, G. A., Grenell, A., Vest, N. A., Moore, K., & Fyfe, E. R. (2023). Links between repeating and growing pattern knowledge and math outcomes in children and adults. *Child Development*, *94*(2), e103–e118. Braasch, J. L., & Goldman, S. R. (2010). The role of prior knowledge in learning from analogies in science texts. *Discourse Processes*, *47*(6), 447–479. Bustamante, A. S., Begolli, K. N., Alvarez-Vargas, D., Bailey, D. H., & Richland, L. E. (2022). Fraction ball: Playful and physically active fraction and decimal learning. *Journal of Educational Psychology*, *114*(6), 1307. Chan, J., & Schunn, C. (2015). The impact of analogies on creative concept generation: Lessons from an in vivo study in engineering design. *Cognitive Science*, *39*(1), 126–155. DeWolf, M., Bassok, M., & Holyoak, K. J. (2016). A set for relational reasoning: Facilitation of algebraic modeling by a fraction task. *Journal of Experimental Child Psychology*, *152*, 351–366. Dumas, D., Schmidt, L. C., & Alexander, P. A. (2016). Predicting creative problem solving in engineering design. *Thinking Skills and Creativity*, *21*, 50–66. Dunbar, K. (1995). How scientists really reason: Scientific reasoning in real-world laboratories. In R. J. Sternberg, & J. Davidson (Eds.), *The nature of insight* (pp. 365–396). MIT Press. Farrington-Flint, L., Canobi, K. H., Woor, C., & Faulkner, D. (2007). The role of relational reasoning in children’s addition concepts. *British Journal of Developmental Psychology*, *25*, 227–246. Goswami, U., & Mead, F. (1992). Onset and rime awareness and analogies in reading. *Reading Research Quarterly*, *27*(2), 153–162. Halpern, D. F., Hansen, C., & Riefer, D. (1990). Analogies as an aid to understanding and memory. *Journal of Educational Psychology*, *82*(2), 298–306. Hansen, J., & Richland, L. E. (2020). Teaching and learning science through multiple representations: Intuitions and executive functions. *CBE—Life Sciences Education*, *19*(4), ar61. Klein, P. D., Piacente-Cimini, S., & Williams, L. A. (2007). The role of writing in learning from analogies. *Learning and Instruction*, *17*(6), 595–611. Kok, E. M., de Bruin, A. B., Robben, S. G., & van Merriënboer, J. J. (2013). Learning radiological appearances of diseases: Does comparison help? *Learning and Instruction*, *23*, 90–97. Matlen, B. J., Gentner, D., & Franconeri, S. (2020). Spatial alignment facilitates visual comparison. *Journal of Experimental Psychology: Human Perception and Performance*, *46*(5), 443–457. Mayer, R. E. (Ed.). (2014). *The Cambridge handbook of multimedia learning* (2nd ed.). Cambridge University Press. Murphy, P. K., Firetto, C. M., & Greene, J. A. (2017). Enriching students’ scientific thinking through relational reasoning: Seeking evidence in texts, tasks, and talk. *Educational Psychology Review*, *29*, 105–117. Novick, L. R., & Holyoak, K. J. (1991). Mathematical problem solving by analogy. *Journal of Experimental Psychology: Learning, Memory, and Cognition*, *17*, 398–415. Richland, L. E., Holyoak, K. J., & Stigler, J. W. (2004). Analogy use in eighth-grade mathematics classrooms. *Cognition and Instruction*, *22*(1), 37–60. Spiro, R. J., Feltovich, R. L., & Anderson, D. K. (1988). *Multiple analogies for complex concepts: Antidotes for analogy-induced misconception in advanced knowledge acquisition*. Center for the Study of Reading Technical Report (No. 439). University of Illinois at Urbana–Champaign. White, C. S. (1998). The relationship between young children’s analogical reasoning and mathematical learning. *Mathematical Cognition*, *4*(2), 103–123. Zhao, H., Alexander, P. A., & Sun, Y. (2021). Relational reasoning’s contributions to mathematical thinking and performance in Chinese elementary and middle-school students. *Journal of Educational Psychology*, *113*(2), 279–292. Zook, K. B., & Di Vesta, F. J. (1991). Effects of analogical processes on learning and misrepresentation. *Educational Psychology Review*, *3*(1), 41–72.
Relational reasoning in expertise development	Chi, M. T. H., Feltovich, P. J., & Glaser, R. (1981). Categorization and representation of physics problems by experts and novices. *Cognitive Science*, *5*, 121–152. Goldwater, M. B., & Gentner, D. (2015). On the acquisition of abstract knowledge: Structural alignment and explication in learning causal system categories. *Cognition*, *137*, 137–153. Goldwater, M. B., Gentner, D., LaDue, N. D., & Libarkin, J. C. (2021). Analogy generation in science experts and novices. *Cognitive Science*, *45*(9), e13036. Rottman, B. M., Gentner, D., & Goldwater, M. B. (2012). Causal systems categories: Differences in novice and expert categorization of causal phenomena. *Cognitive Science*, *36*(5), 919–932.
Development of relational reasoning/higher-order thinking	Frausel, R. R., Richland, L. E., Levine, S. C., & Goldin-Meadow, S. (2021). Personal narrative as a “breeding ground” for higher-order thinking talk in early parent–child interactions. *Developmental Psychology*, *57*(4), 519–533. Frausel, R. R., Silvey, C., Freeman, C., Dowling, N., Richland, L. E., Levine, S. C., & Goldin-Meadow, S. (2020). The origins of higher-order thinking lie in children’s spontaneous talk across the preschool years. *Cognition*, *200*, 104274. Frausel, R. R., Vollman, E., Muzard, A., Richland, L. E., Goldin-Meadow, S., & Levine, S. C. (2022). Developmental trajectories of early higher-order thinking talk differ for typically developing children and children with unilateral brain injuries. *Mind, Brain, and Education*, *16*(2), 153–166. Gentner, D. (1977). Children’s performance on a spatial analogies task. *Child Development, 48*, 1034–1039. Gentner, D. (1988). Metaphor as structure mapping: The relational shift. *Child Development*, 47–59. Goswami, U. (2002). Inductive and deductive reasoning. In U. Goswami (Ed.), *Blackwell handbook of childhood cognitive development* (pp. 282–302). Blackwell. Kotovsky, L., & Gentner, D. (1996). Comparison and categorization in the development of relational similarity. *Child Development*, *67*(6), 2797–2822. Krawczyk, D. C. (2012). The cognition and neuroscience of relational reasoning. *Brain Research*, *1428*, 13–23. Krawczyk, D. C., Hanten, G., Wilde, E. A., Li, X., Schnelle, K. P., Merkley, T. L., & Levin, H. S. (2010). Deficits in analogical reasoning in adolescents with traumatic brain injury. *Frontiers in Human Neuroscience*, *4*, 62. Krawczyk, D. C., Morrison, R. G., Viskontas, I. V., Holyoak, K. J., Chow, T. W., Mendez, M. F., & Knowlton, B. J. (2008). Distraction during relational reasoning. *Neuropsychologia*, *46*(7), 2020–2032. Morrison, R. G., Krawczyk, D. C., Holyoak, K. J., Hummel, J. E., Chow, T. W., Miller, B. L., & Knowlton, B. J. (2004). A neurocomputational model of analogical reasoning. *Journal of Cognitive Neuroscience*, *16*(2), 260–271. Pruden, S. M., Levine, S. C., & Huttenlocher, J. (2011). Children’s spatial thinking. *Developmental Science*, *14*(6), 1417–1430. Rattermann, M. J., & Gentner, D. (1998). More evidence for a relational shift in the development of analogy. *Cognitive Development*, *13*(4), 453–478. Richland, L. E., Morrison, R. G., & Holyoak, K. J. (2006). Children’s development of analogical reasoning. *Journal of Experimental Child Psychology*, *94*(3), 249–273. Rowe, M. L., & Goldin-Meadow, S. (2009). Differences in early gesture explain SES disparities. *Science*, *323*(5916), 951–953. Silvey, C., Gentner, D., Richland, L. E., & Goldin-Meadow, S. (2023). Children’s early spontaneous comparisons predict later analogical reasoning skills. *Open Mind*, *7*, 483–509. Waltz, J. A., Knowlton, B. J., Holyoak, K. J., Boone, K. B., Mishkin, F. S., de Menezes Santos, M., & Miller, B. L. (1999). A system for relational reasoning in human prefrontal cortex. *Psychological Science*, *10*(2), 119–125.
Relational reasoning and explanation	Atkinson, R. K., Derry, S. J., Renkl, A., & Wortham, D. (2000). Learning from examples. *Review of Educational Research*, *70*, 181–214. Chi, M. T. H., De Leeuw, N., Chiu, M. H., & LaVancher, C. (1994). Eliciting self-explanations improves understanding. *Cognitive Science*, *18*(3), 439–477. Lombrozo, T. (2006). The structure and function of explanations. *Trends in Cognitive Sciences*, *10*(10), 464–470. Lombrozo, T. (2012). Explanation and abductive inference. In K. J. Holyoak, & R. G. Morrison (Eds.), *The Oxford handbook of thinking and reasoning* (pp. 260–276). Oxford University Press.
Relational reasoning and artificial intelligence	Lu, H., Wu, Y. N., & Holyoak, K. J. (2019). Emergence of analogy from relation learning. *Proceedings of the National Academy of Sciences*, *116*(10), 4176–4181. Webb, T., Holyoak, K. J., & Lu, H. (2023). Emergent analogical reasoning in large language models. *Nature Human Behaviour*, *7*(9), 1526–1541.
Relational mindset	Goldwater, M. B., & Jamrozik, A. (2019). Can a relational mindset boost analogical retrieval? *Cognitive Research: Principles and Implications*, *4*, 1–16. Murphy, A. N., Zheng, Y., Shivaram, A., Vollman, E., & Richland, L. E. (2021). Bias and sensitivity to task constraints. *Journal of Experimental Child Psychology*, *202*, 104981. Simms, N. K., & Richland, L. E. (2019). Generating relations elicits a relational mindset in children. *Cognitive Science*, *43*(10), e12795. Vendetti, M. S., Matlen, B. J., Richland, L. E., & Bunge, S. A. (2015). Analogical reasoning in the classroom. *Mind, Brain, and Education*, *9*(2), 100–106. Vendetti, M. S., Wu, A., & Holyoak, K. J. (2014). Far-out thinking. *Psychological Science*, *25*(3), 1–6. Walker, C. M., Hubachek, S. Q., & Vendetti, M. S. (2018). Achieving abstraction. *Developmental Psychology*, *54*(10), 1833–1841. Zhao, H., Richland, L. E., Vollman, E., Lerner, B. S., Yeung, N. A., & Wong, J. (2025). Individual differences in attention to analogical relations. *Journal of Cognition and Development*. Zhao, H., Rose, E., & Richland, L. E. (2025). Priming multiplicative thinking across tasks. *SRCD Annual Conference*.
Analogy-based teaching practices	Alfieri, L., Nokes-Malach, T. J., & Schunn, C. D. (2013). Learning through case comparisons. *Educational Psychologist*, *48*, 87–113. Alibali, M. W., Nathan, M. J., Wolfgram, M. S., Church, R. B., Jacobs, S. A., Johnson Martinez, C., & Knuth, E. J. (2014). How teachers link ideas in mathematics instruction. *Cognition and Instruction*, *32*(1), 65–100. Begolli, K. N., Richland, L. E., Jaeggi, S. M., Lyons, E. M., Klostermann, E. C., & Matlen, B. J. (2018). Executive function in learning mathematics by comparison. *Thinking & Reasoning*, *24*(2), 280–313. Blessing, S. B., & Ross, B. H. (1996). The effect of problem content on categorization and problem solving. *Journal of Experimental Psychology: Learning, Memory, and Cognition*, *22*, 792–810. Durkin, K., Rittle-Johnson, B., Star, J. R., & Loehr, A. (2023). Comparing and discussing multiple strategies. *Journal of Experimental Education*, *91*(1), 1–19. Richland, L. E., & McDonough, I. M. (2010). Learning by analogy. *Contemporary Educational Psychology*, *35*(1), 28–43. Richland, L. E., Zur, O., & Holyoak, K. J. (2007). Cognitive supports for analogies in the mathematics classroom. *Science*, *316*(5828), 1128–1129. Rittle-Johnson, B., & Star, J. R. (2007). Does comparing solution methods facilitate knowledge? *Journal of Educational Psychology*, *99*(3), 561–574. Rittle-Johnson, B., Star, J. R., & Durkin, K. (2009). The importance of prior knowledge when comparing examples. *Journal of Educational Psychology*, *101*(4), 836–852. Rittle-Johnson, B., Star, J. R., & Durkin, K. (2017). The power of comparison in mathematics instruction. In *Mathematical cognition and learning* (Vol. 3). Elsevier. Star, J. R., & Rittle-Johnson, B. (2008). Flexibility in problem solving. *Learning and Instruction*, *18*(6), 565–579. Star, J. R., Pollack, C., Durkin, K., Rittle-Johnson, B., Lynch, K., Newton, K., & Gogolen, C. (2015). Learning from comparison in algebra. *Contemporary Educational Psychology*, *40*, 41–54. Williams, T., Ferraro, D., Roey, S., Brenwald, S., Kastberg, D., Jocelyn, L., & Stearns, P. (2009). *TIMSS 2007 U.S. technical report and user guide.* NCES.
